# Beta-amyloid induces apoptosis of neuronal cells by inhibition of the Arg/N-end rule pathway proteolytic activity

**DOI:** 10.18632/aging.102177

**Published:** 2019-08-24

**Authors:** Olga I. Kechko, Irina Yu. Petrushanko, Christopher S. Brower, Alexei A. Adzhubei, Alexey A. Moskalev, Konstantin I. Piatkov, Vladimir A. Mitkevich, Alexander A. Makarov

**Affiliations:** 1 Engelhardt Institute of Molecular Biology, Russian Academy of Sciences, Moscow 119991, Russia; 2 Department of Biology, Texas Woman’s University, Denton, TX 76204, USA; 3 Institute of Biology, Komi Science Center, Russian Academy of Sciences, Syktyvkar 167000, Russia; 4 Moscow Institute of Physics and Technology, Dolgoprudny, Moscow Region 141701, Russia; 5 Center of Life Sciences, Skolkovo Institute of Science and Technology, Moscow 121205, Russia

**Keywords:** age-related disease, Alzheimer’s disease, apoptosis, Ate1, protein degradation

## Abstract

Alzheimer’s disease (AD) is accompanied by the dysfunction of intracellular protein homeostasis systems, in particular the ubiquitin-proteasome system (UPS). Beta-amyloid peptide (Aβ), which is involved in the processes of neurodegeneration in AD, is a substrate of this system, however its effect on UPS activity is still poorly explored. Here we found that Aβ peptides inhibited the proteolytic activity of the antiapoptotic Arg/N-end rule pathway that is a part of UPS. We identified arginyltransferase Ate1 as a specific component of the Arg/N-end rule pathway targeted by Aβs. Aβ bearing the familial English H6R mutation, known to cause early-onset AD, had an even greater inhibitory effect on protein degradation through the Arg/N-end rule pathway than intact Aβ. This effect was associated with a significant decrease in Ate1-1 and Ate1-3 catalytic activity. We also found that the loss of Ate1 in neuroblastoma Neuro-2a cells eliminated the apoptosis-inducing effects of Aβ peptides. Together, our results show that the apoptotic effect of Aβ peptides is linked to their impairment of Ate1 catalytic activity leading to suppression of the Arg/N-end rule pathway proteolytic activity and ultimately cell death.

## Introduction

Alzheimer’s disease (AD) is a neurological disorder caused by the generalized and progressive death of neurons. It is the most widespread neurodegenerative disease of humankind and is accompanied by the accumulation of damaged, misfolded or otherwise abnormal proteins. The main neuromorphological features of AD are amyloid plaques composed largely of the beta-amyloid peptide (Aβ), and neurofibrillary tangles of the microtubule binding protein tau [1–3]. The accumulation of pathological proteins causing neuronal death is associated with the dysfunction and/or dysregulation of intracellular protein homeostasis systems [4–6]. The first such system to be impaired is the ubiquitin-proteasome system (UPS). Impairments of the UPS initiate learning and memory deficits in Aβ transgenic animals that is associated with AD development [4]. The UPS marks damaged or misfolded proteins by the covalent attachment of polyubiquitin. Ubiquitylated proteins are then targeted for degradation by the proteasome, a large multiprotein complex possessing both proteasome-associated trypsin- and chymotrypsin-like endoproteolytic activities. Previously, it was shown that the chymotrypsin-like activity of the proteasome was reduced in AD brains [7,8]. Impairment of proteasomal activity in AD is linked to Aβ, a highly amyloidogenic, 39-43 amino acid polypeptide that has been shown to induce apoptosis leading to neuronal cell death [9,10]. Although it is unclear exactly how Aβ leads to apoptosis, Aβ_42_ was shown to directly decrease chymotrypsin-like activity of proteasome [11,12]. However, additional studies in both animal and cellular models of AD, as well as *in vitro,* reported a variety of Aβ effects on the proteasome ranging from decreased to increased and even unchanged activity [13–15]. Therefore, a greater understanding of the relationship between Aβ and the UPS is needed in order to appreciate how Aβ is toxic to cells and how it induces apoptosis in neurons.

The N-end rule pathway is a part of the UPS and plays critical role in proteolytic signaling and protein-quality control. This pathway recognizes proteins and polypeptides containing N-terminal degradation signals (termed N-degrons) and facilitates their polyubiquitylation thereby facilitating their degradation by the proteasome [16–18]. The main determinant of an N-degron is a destabilizing N-terminal residue. N-degrons are recognized by specific E3 ubiquitin ligases of the N-end rule pathway. In mammals this pathway consists of the two branches, the Ac/N-end rule pathway, which degrades proteins bearing acetylated N-terminal amino acids [19–21], and the Arg/N-end rule pathway, which degrades proteins bearing non-acetylated N-terminal arginine, lysine, histidine, leucine, phenylalanine, tryptophan, tyrosine, isoleucine or methionine (if followed by a hydrophobic amino acid) [17,22–24]. A number of additional N-terminal amino acids are destabilizing as well but require their prior modifications before recognition by N-end rule E3 ligases. N-terminal amidohydrolases catalyze the conversion of asparagine and glutamine into aspartate and glutamate, respectively [25–27]. Proteins bearing N-terminal aspartate, glutamate and oxidized cysteine are N-terminally arginylated by the arginyl-tRNA-protein transferase (Ate1) [28–30]. Protein arginylation is a two-step reaction. Initially, tRNA is charged with arginine by aminoacyl-tRNA synthetase (RS) in a manner that requires ATP. Arginine is then transferred from tRNA^Arg^ to the substrate by R-transferase Ate1 [31,32]. Previously, Brower, *et al.* found that Ate1 was capable of arginylating Aβ peptides and that arginylated Aβ_42_ is destroyed by proteasome [24]. However, the role of Ate1 and the Arg/N-end rule pathway in Aβ-associated neurotoxicity has not been fully explored. Additionally, Piatkov, *et al.* found that the Arg/N-end rule pathway counteracts apoptotic cell death by degrading proapoptotic protein fragments generated by caspase activation. They showed that caspases were capable of inactivating Ate1 as well as additional components of the Arg/N-end rule pathway suggesting a mutual suppression between proapoptotic signaling and the N-end rule pathway [23]. We hypothesize that the apoptosis-inducing effect of Aβ is mediated through the inhibition of the Arg/N-end rule pathway of the UPS.

To test this hypothesis, we examined the Arg/N-end rule pathway in the presence of Aβ_42_ or its pathogenic mutant carrying the ‘English’ mutation H6R (H6R-Aβ_42_), which is more amyloidogenic and is associated with early-onset AD [33–35]. We found that the apoptotic effects of Aβ peptides are associated with decreased Atel activity and inhibition of protein degradation via the Arg/N-end rule pathway.

## RESULTS

### Aβ peptides inhibit the proteolytic activity of the Arg/N-end rule pathway

**Figure 1:**
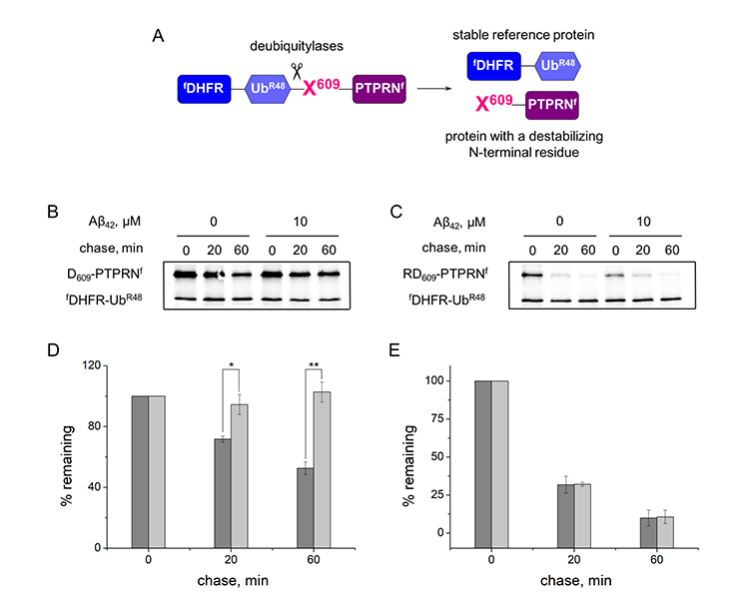
**Inhibition effect of Aβ_42_ (10 μM) on the proteolytic activity of the Arg/N-end rule pathway.** (**A**) Diagram of the ^f^DHFR-Ub^R48^-X_609_-PTPRN^f^ (X = Asp, Arg-Asp) fusion. Co-translational cleavage of the fusion by deubiquitylases produces a test protein X_609_-PTPRN^f^ and a stable ’reference’ protein ^f^DHFR-Ub^R48^ at the initially equimolar ratio. (**B**) Degradation of Asp_609_-PTPRN^f^ in reticulocyte lysate in the presence or absence of Aβ_42_. Asp_609_-PTPRN^f^ was expressed in reticulocyte lysate and co-translationally labeled with ^35^S-Met for 30 min at 30°C in the presence or absence of Aβ_42_, followed by a chase, immunoprecipitation with anti-flag M2 antibody, SDS-PAGE, and autoradiography. (**C**) Same as (B) but with Arg-Asp_609_-PTPRN^f^ fragment. (**D**) Quantification of (B). The level of Asp_609_-PTPRN^f^ was normalized on the level of ^f^DHFR-Ub^R48^. The level of Asp_609_-PTPRN^f^ detected immediately after stopping of protein expression in reticulocyte lysate (0 min chase) was taken as 100%. "% remaining" is the level of non-degraded Asp_609_-PTPRN^f^ at shown time points after stopping of protein expression. The absence of Aβ_42_ - dark-gray column; the presence of Aβ_42_ – light-gray column. (**E**) Quantification of (C). Each value is the mean ± SD of at least three independent experiments; *p < 0.01, **p < 0.001.

To evaluate the ability of Aβ_42_ and H6R-Aβ_42_ to modulate activity of the Arg/N-end rule pathway the ubiquitin reference technique (URT) was used [36]. This technique is based on the comparison of degradation rates of a test protein with a destabilizing N-terminal residue and a reference protein, which is not recognized by components of the N-end rule pathway. In this study we utilized ^f^DHFR-Ub^R48^, a flag-tagged derivative of the mouse dihydrofolate reductase as a reference protein, and PTPRN (Ica512) fragment as a test protein. As was shown earlier, calpain-generated Lys_609_-PTPRN fragment is a short-lived substrate of the Arg/N-end rule pathway [23]. In the URT-based pulse-chase assays the ^f^DHFR-Ub^R48^-X_609_-PTPRN^f^ (X = Asp, Arg-Asp) fusion protein is co-translationally cleaved by deubiquitilases, yielding equimolar quantities of the test and reference proteins (Fig. 1A). The labeled test protein was quantified by measuring its level relative to the level of a stable reference at the same time point.

The N-terminal amino acid residues of the test protein X_609_-PTPRN^f^ were Asp, which required Ate1 for degradation by the Arg/N-end rule pathway, and Arg, which bypasses the need for Ate1 and is directly recognized by the Arg/N-end rule E3 ubiquitin ligases [17]. In the absence of amyloid peptides both Asp-PTPRN^f^ and Arg-PTPRN^f^ are rapidly degraded in the URT-based pulse-chase assays (initial posttranslational t_1/2_ ~60 min for Asp_609_-PTPRN^f^ and ~14 min for Arg-Asp-PTPRN^f^) (Fig. 1B, C, D, E). In the presence of Aβ_42_ however, degradation of Asp-PTPRN^f^ was completely inhibited whereas Arg-PTPRN^f^ was unchanged (Fig. 1B, C, D, E), which suggests that Aβ_42_ disrupts the arginylation of proteins.

**Figure 2:**
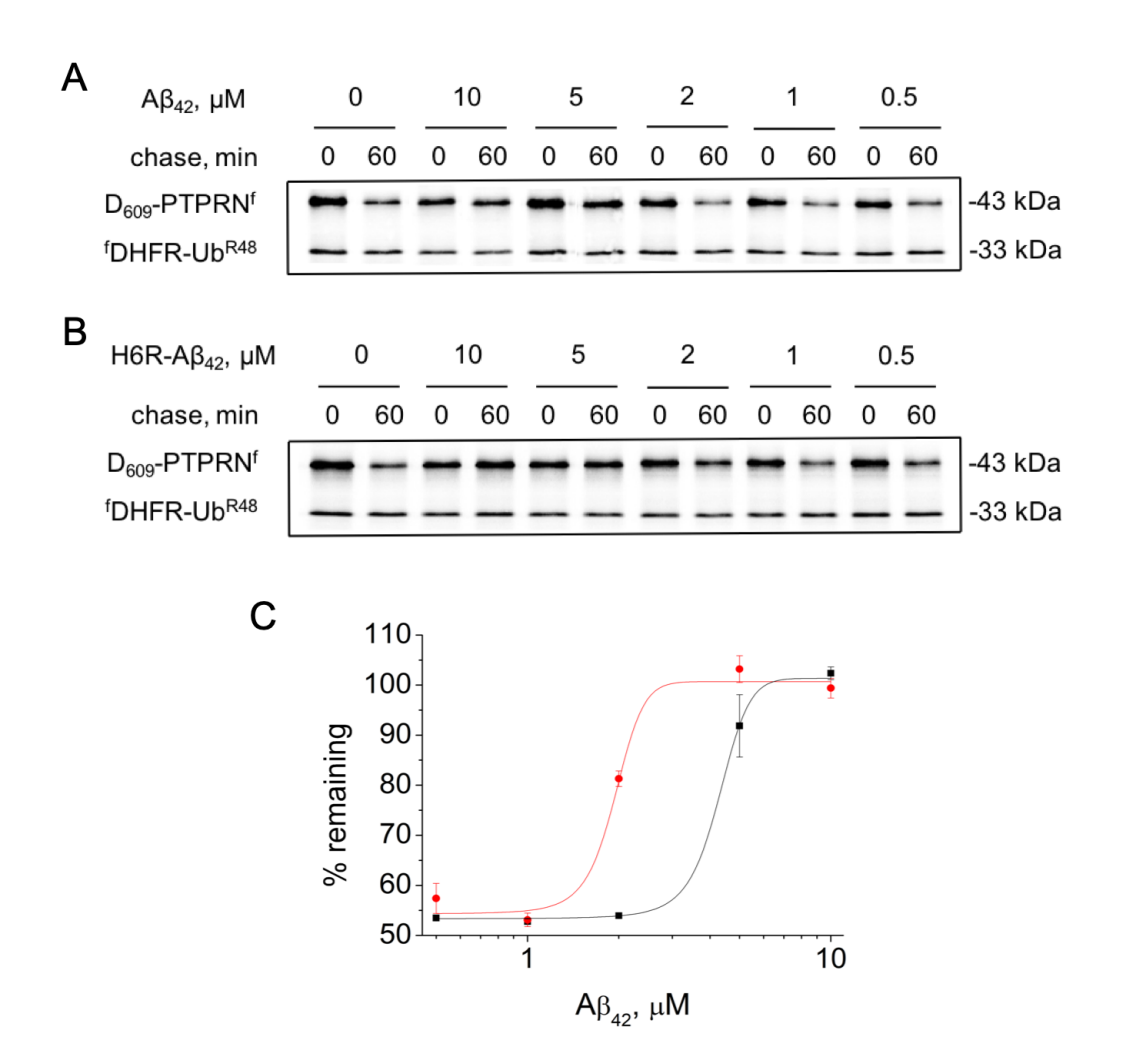
**Effect of different concentrations of amyloid peptides on the proteolytic activity of the Arg/N-end rule pathway.** Degradation of ^35^S-methionine labeled Asp_609_-PTPRN^f^ in the presence of Aβ_42_ (**A**) or H6R-Aβ_42_ (**B**) was assayed as described in the legend to Figure 1B. (**C**) – quantification of (A) and (B) at 60 min chase (Aβ_42_ – black squares, H6R-Aβ_42_ – red circles). Each value is the mean expressed as a percentage of the level of Asp_609_-PTPRN^f^ at 0 min chase ± SD of at least three independent experiments.

H6R-Aβ_42_ had significantly stronger inhibitory effects on the degradation of Asp_609_-PTPRN^f^ compared to Aβ_42_. Thus IC_50_ (half maximal inhibitory concentration) for H6R-Aβ_42_ is 1.83 ± 0.56 μM compared to 4.27 ± 0.15 μM for Aβ_42_ (Fig. 2).

### Aβ_42_ interacts with the R-transferase Ate1

**Figure 3:**
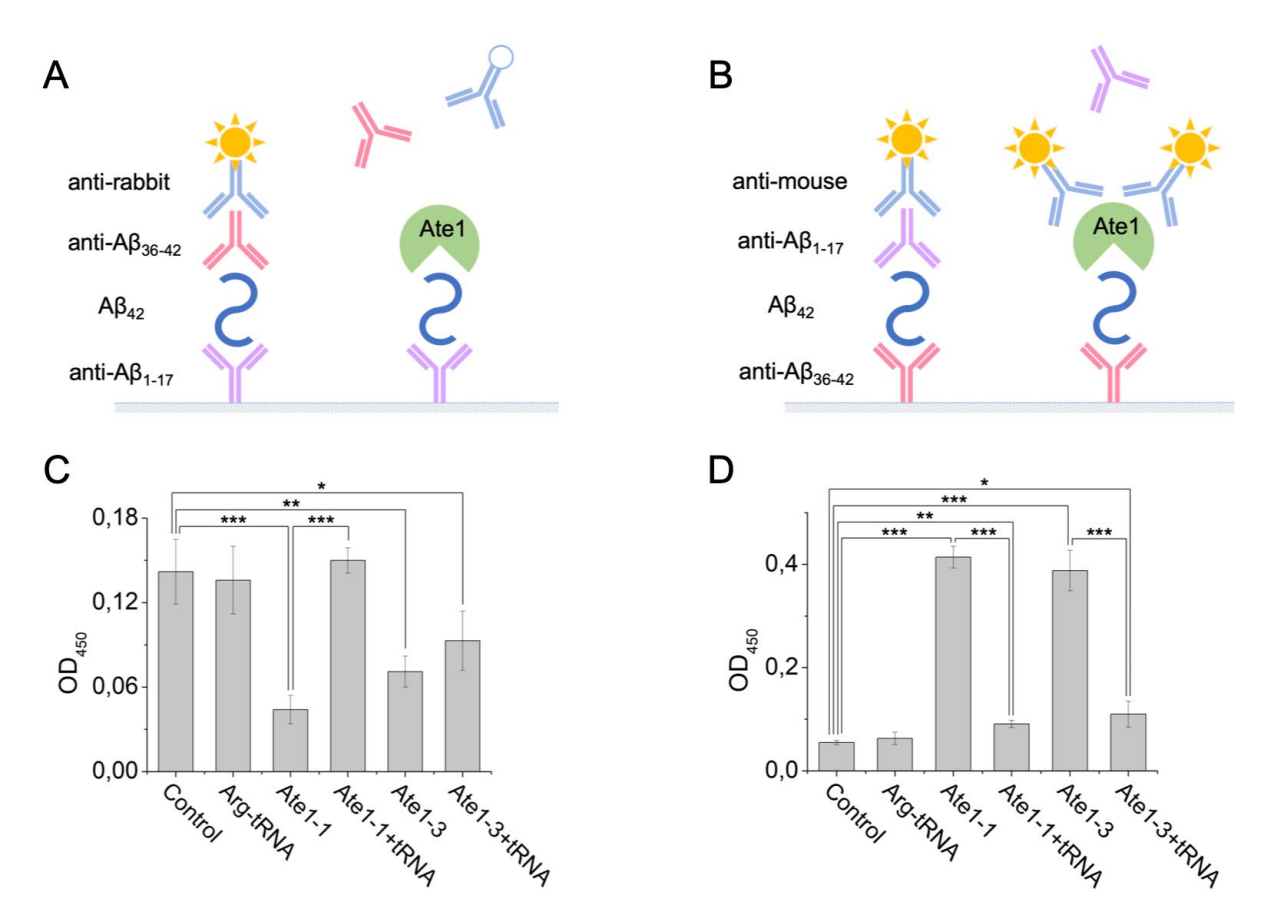
**Interaction of Aβ_42_, tRNA, and Ate1.** Schematic representation of ELISA assay with immobilized anti-Aβ1-17 antibodies (**A**) or anti-Aβ36-42 antibodies (**B**). (**C**) Detection of tRNA and Ate1 ability to interact with C-terminus of Aβ. (**D**) Same as (C) but with N-terminal region of Aβ. OD_450_ – optical density measured at 450 nm. Each value is the mean ± SD of at least four independent experiments; *p < 0.04, **p < 0.01, ***p < 0.001.

It was shown earlier that different isoforms of Ate1 can arginylate Aβ_42_ with unequal effectiveness [24]. In this study we used the Ate1-3 and Ate1-1 isoforms. The Ate1-3 isoform is more effective in arginylating the peptide than Ate1-1 isoform. Interaction of Aβ_42_ with tRNA and Ate1 was probed using the sandwich ELISA method (Fig. 3A). A pair of antibodies was used that recognize different segments of Aβ_42_, the N-terminal Aβ_1-17_ and C-terminal Aβ_36-42_. tRNA, Ate1 and their combination were added to Aβ_42_ bound to immobilized anti-Aβ_1-17_ antibodies. Interaction of any component of the reaction mixture with Aβ_42_ blocks binding of the anti-Aβ_36-42_ antibodies to the C-terminal segment of the peptide, as well as the secondary anti-rabbit antibodies, leading to decreased spectrophotometric signal. Addition of Ate1-1 or Ate1-3 significantly reduced optical density of the solution, indicating that the interaction of Aβ_42_ with detection antibodies was blocked by Ate1 (Fig. 3C). In contrast, tRNA had no impact on the interaction of Aβ_42_ with detection antibodies. The concurrent addition of tRNA and Ate1-1 did not change the signal compared to control, however the combination of tRNA and Ate1-3 blocked the interaction of Aβ_42_ with detection antibodies similarly to Ate1-3 alone (Fig. 3C).

Interaction of the N-terminal region of Aβ_42_ with Ate1 isoforms and tRNA was studied using anti-Aβ_36-42_ as capture antibodies, and anti-Aβ_1-17_ as detection antibodies (Fig. 3B). tRNA did not affect the interaction of Aβ_42_ with antibodies. We revealed cross-reactivity of Ate1 and secondary anti-mouse antibodies (Suppl. Fig. 1). This effect was used to detect the binding of Ate1 with Aβ_42_. Addition of Ate1-1 or Ate1-3 significantly enhanced optical density of the solution, indicating that Ate1 isoforms interact with N-terminus of Aβ_42_ (Fig. 3D). tRNA blocked the interaction of the amyloid peptide with both Ate1 isoforms.

### Aβ peptides decrease enzymatic activity of Ate1

We examined the kinetic parameters of protein arginylation using isothermal titration calorimetry (ITC). ATP hydrolysis carried out by RS is an exothermic reaction (Suppl. Fig. 2A, B). Rate data for this reaction were fitted to the Michaelis-Menten equation, and kinetic constants were obtained (Table 1). Amyloid peptides were added to the calorimetric cell as a competitive inhibitor (Suppl. Fig. 2C, D, E, F). The K_I_ of RS for Aβ_42_ and H6R-Аβ_42_ were 157 and 161 μM, respectively (Table 1).

**Table 1:** Inhibition effect of Аβ peptides on the enzyme kinetics of RS.

**Inhibitior**	**K_m_ (µМ)**	**k_cat_ (s^-1^)**	**K_I_ (µМ)**	**ΔH (kcal/mol)**
**-**	35.7 ± 1.2	0.56 ± 0.09	-	-3.16
**Аβ_42_**	-	-	157.0 ± 5.2	-1.48
**H6R-Аβ_42_**	-	-	161.0 ± 1.3	-2.77

Comparison of the enzymatic kinetic parameters of Ate1 isoforms were carried out for its well-known substrate BSA [31,32,37] (Suppl. Fig. 3A, B; 4A, B), Аβ_42_ (Suppl. Fig. 3C, D; 4C, D) and H6R-Аβ_42_ (Suppl. Fig. 3E, F; 4E, F). For Аβ_42_ the values of k_cat_ for Ate1-1 and Ate1-3 were ~6- and ~13-fold lower than for BSA, respectively (Table 2). At the same time, K_m_ values for Аβ_42_ decreased ~8-fold for Ate1-1 and ~11-fold for Ate1-3 compared to BSA. Arginylation of H6R-Аβ_42_ proceeded at an even slower rate (Table 2), with k_cat_ values for Ate1-1 and Ate1-3 decreasing ~14-fold and ~55-fold, respectively; and K_m_ values decreasing ~14-fold and ~23-fold, respectively, relative to BSA.

**Table 2:** Parameters of Ate1 enzyme kinetics.

**Substrate**	**Enzyme**	**K_m_ (µМ)**	**k_cat_ (s^-1^)**	**ΔH (kcal/mol)**
**BSA**	**Ate1-1**	33.2 ± 4.0	6.6 ± 0.6	5.87
**Аβ_42_**		4.3 ± 0.5	1.07 ± 0.09	-10.4
**H6R-Аβ_42_**		2.4 ± 0.5	0.46 ± 0.05	-7.85
**BSA**	**Ate1-3**	52.5 ± 11.8	9.3 ± 1.7	5.78
**Аβ_42_**		4.6 ± 0.3	0.70 ± 0.04	-9.77
**H6R-Аβ_42_**		2.3 ± 0.3	0.17 ± 0.03	-7.25

### Ate1 is required for apoptosis induced by amyloid peptides

**Figure 4:**
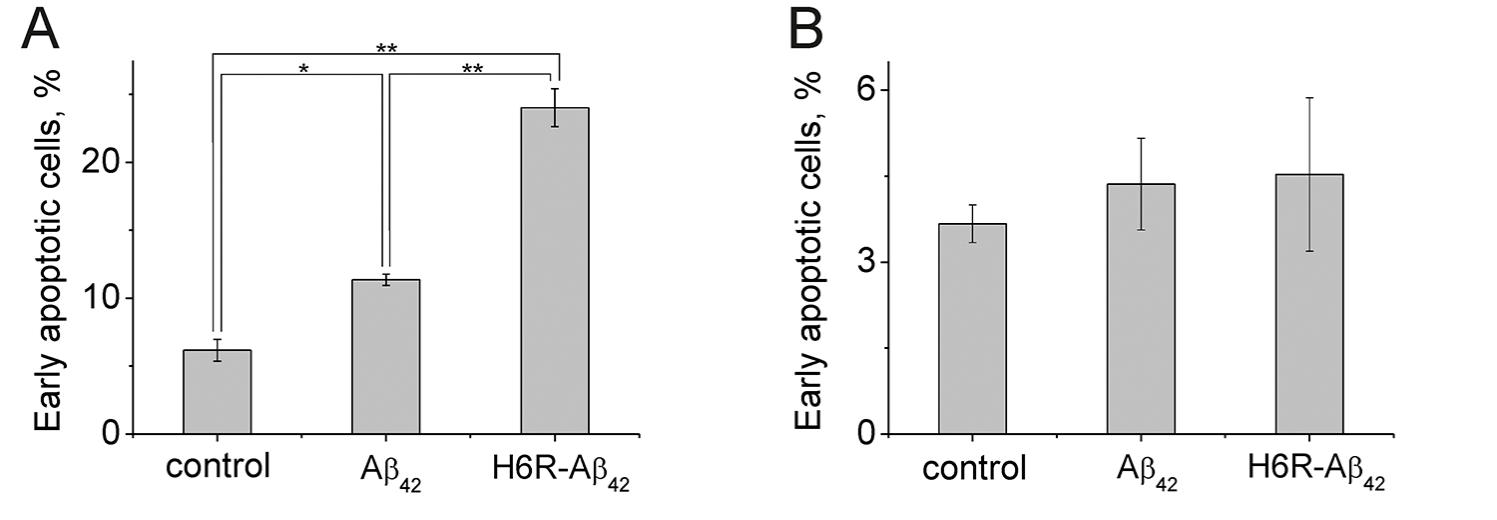
Apoptotic effects of Aβ peptides (10 µM, 20 h) on Neuro-2a (**A**) cells and Ate1 knockout Neuro-2a cells (**B**). The Annexin-V positive and PI negative cells were considered early apoptotic. Each value is expressed as a percentage of the total number of cells ± SD. The experiments were performed three times in triplicates; *p < 0.01, **p < 0.001.

The apoptotic effect of Aβ peptides on differentiated mouse neuroblastoma Neuro-2a cells was assessed by flow cytometry after 20 hours of incubation of cells with peptides. We found that the percentage of early apoptotic wild type Neuro-2a cells (Annexin V^+^ propidium iodide^-^) increased ~2-fold (from ~6% to ~12%) in the presence of Аβ_42_ (Fig. 4A). H6R-Aβ_42_ had an even greater effect as the number of early apoptotic wild type cells increased ~4-fold relative to untreated cells (from ~6% to ~24%) (Fig. 4A) the number of late apoptotic cells increased 3-fold relative to control (Suppl. Fig. 5A). At the same time presence of Aβs results in increase the number of necrotic cells in population (Suppl. Fig. 5C). Consistent with the apoptotic effects of amyloid peptides being mediated through Ate1, we found no increase in percentage of early and late apoptotic Ate1-lacking Neuro-2a cells treated with either Aβ_42_ or H6R-Aβ_42_ (Fig. 4B, Suppl. Fig. 5B). In the presence of H6R-Aβ_42_ the percentage of necrotic Ate1-lacking Neuro-2a cells increased, which may be due to its nonspecific membrane-damaging effect (Suppl. Fig. 5D).

## DISCUSSION

AD results from the progressive dysfunction and death of neurons associated with the amyloidogenic peptide, Aβ. A number of familial mutations of Aβ enhance its oligomerization and toxic properties, triggering the development of early-onset AD [38–41]. One of such mutations is the ‘English’ mutation defined by the H6R substitution (H6R-Aβ_42_) [33]. Many studies over the past decade have shown toxicity associated with Aβ in various oligomeric states [9,42–44]. Large aggregates can result in mechanical damage of the cell membrane, leading to disruption of the ion homeostasis and necrosis. Small Aβ oligomers can bind to membrane receptors and/or penetrate cells where they disrupt function of the cellular systems and trigger apoptosis. One major anti-apoptotic systems in cells is the Arg/N-end rule pathway of the ubiquitin proteasome system, which degrades caspase-generated pro-apoptotic protein fragments. Indeed, even a partial inhibition of this pathway leads to the accumulation of pro-apoptotic protein fragments, an increase in caspase activation by positive feedback, and increased apoptosis [23]. In this study, we found that Aβ, and especially H6R-Aβ_42,_ induces cellular apoptosis by inhibiting protein arginylation by Ate1 and subsequent protein degradation through the Arg/N-end rule pathway.

The Arg/N-end rule dependent degradation of proteins (or protein fragments) bearing N-terminal Asp, Glu, or oxidized Cys requires their conjugation, by one of the *ATE1*-encoded isoforms of the arginyl-tRNA-protein transferase (Ate1) to arginine [17,45–47]. This reaction also requires tRNA^Arg^ formed by aminoacyl-tRNA synthetases (RS). Mammals have at least six Ate1 isoforms produced through alternative splicing that differ by 1 and 7 exons [45]. The level of Ate1 isoforms significantly varies in different tissues and intracellular compartments, suggesting that specific isoforms may have distinct functions [46,47]. All isoforms have different activity and substrate specificity [31,46,47]. It was shown that Ate1-2 and Ate1-3 arginylate Aβ_42_ with significantly higher efficiency than Ate1-1 and Ate1-4 [24].

We found that the amyloid peptides Aβ_42_ and H6R-Aβ_42_ had little effect on RS activity (Suppl. Fig. 2, Table 2) but were capable of binding the Ate1 isoforms 1-1 and 1-3 (Fig. 3B). We also found that the presence of tRNA decreased Ate1-1, but not Ate1-3 binding to Aβ_42_ (Fig. 3B). This may underlie the differences between Ate1-1 and Ate1-3 in their abilities to arginylate Aβ_42_ [24]. We also found that Aβ_42_ and H6R-Aβ_42_ have a higher affinity for Ate1 than BSA, a well-characterized substrate of Ate1 [31,32,37] (Suppl. Fig. 3C, D, E, F, Fig. 4C, D, E, F, and Table 2). At the same time k_cat_ of Aβs arginylation was significantly lower than for BSA (Table 2). Finally, we found that Aβ peptides inhibited the degradation of substrate proteins bearing N-terminal Asp but had no effect on the degradation of proteins bearing primary destabilizing N-terminal amino acids directly recognized by E3 ubiquitin ligases of the Arg/N-end rule pathway (e.g. Arg). Collectively, these data indicate that amyloid peptides compete with natural Ate1 substrates resulting in their decreased arginylation and increased stability. In the case of pro-apoptotic protein fragments that require N-terminal arginylation for degradation by the Arg/N-end rule pathway, such competition by amyloid peptides increases the pro-apoptotic signal within cells. In support of this conclusion, using a genetic approach, we found that Aβ peptides induced the apoptosis of differentiated wild type Neuro-2a cells but not Neuro-2a cells in which Ate1 was ablated by Crispr/Cas9.

Apart from protein degradation by the Arg/N-end rule pathway, post-translational arginylation has been shown to be involved in a number of cellular processes including the modulation of the cell cytoskeleton [48,49], regeneration of neural tissue in lesions [50–52], G-protein signaling [53,54], and angiogenesis [55,56]. Arginylation also plays a protective role in aging [57], stress response regulation [58] and in the prevention of protein aggregation in neurodegeneration [59]. Recently, N-terminal arginylation by Ate1 was shown to signal protein degradation via the autophagy system [60–62] as well. Dysfunction of UPS leads to accumulation of misfolded or damaged proteins in cells. This stimulates translocation of endoplasmic reticulum chaperones, such as BiP, into the cytoplasm where they bind unwanted proteins. Their N-terminal arginylation mediated by Ate1 activates autophagic adaptor p62/STQSM/Sequestosome-1, promoting autophagic flux and lysosomal degradation [60,62,63]. As such, a decrease in protein arginylation by Ate1 activity caused by amyloid peptides may affect a wide array of cellular processes leading to apoptosis.

In conclusion, we suggest that the following succession of events takes place in the cell affected by amyloid peptides. Pathological processes invoked by AD lead to the accumulation of Aβ in the cells [64–68]. When a critical concentration of Aβ is reached, Ate1 function is disrupted, leading to the stabilization of misfolded and toxic proteins, including pro-apoptotic protein fragments otherwise degraded by the Arg/N-end rule pathway, triggering apoptosis leading to neuronal cell death.

## MATERIALS AND METHODS

### Plasmids and primers

43-kDa mouse X_609_-PTPRN (X=Asp, Arg-Asp) fragment was amplified by polymerase chain reaction (PCR) using following primers: AAAAACCGCGGAGGAGATGAGCGCCTGGCAGCGCTGGGGC and TTTTAATCGATCTGGGGCAGGGCCTTGAGGAT for Asp_609_-PTPRN; AAAAACCGCGGAGGACGTGATGAGCGCCTGGCAGCGCTGGGGC and TTTTAATCGATCTGGGGCAGGGCCTTGAGGAT for Arg-Asp_609_-Ica512. Encyclo polymerase (Evrogen) was used for PCR. The resulting PCR products were cut with SacII/ClaI and cloned into SacII/ClaI-cut pKP496 [36]. Turbo *E. сoli* (NEB) was used for cloning and maintaining plasmids. Sequences of all constructed plasmids were verified by DNA sequencing. Plasmids pCB407 and pCB409 encoding Ate1 isoforms were described previously [69].

### Preparation of Aβ peptides

Synthetic peptide Aβ_42_: [H_2_N]-DAEFRHDSGYEVHHQKLVFFAEDVGSNKGAIIGLMVGGVVIA-[COOH] and its mutant H6R-Aβ_42_: [H_2_N]-DAEFRRDSGYEVHHQKLVFFAEDVGSNKGAIIGLMVGGVVIA-[COOH] were purchased from Biopeptide. Monomerization of Aβ peptides was performed as described previously [70]. Briefly, chilled hexafluoroisopropanol (HFIP, Fluka) was added to solid Aβ peptide to a concentration of 1 mM and incubated for 60 min at room temperature. Then this solution was put on ice for 10 min and aliquoted into non-siliconized microcentrifuge tubes (0.23 mg peptide per tube). HFIP evaporated overnight in the hood at room temperature. Peptide in the tubes was dried under vacuum using Eppendorf Concentrator 5301 to remove traces of HFIP. Dried peptide was stored at -80°C. 5 mM peptide stock solution was prepared by adding 10 μl of 100% anhydrous DMSO (Merck) to 0.23 mg peptide and incubating for 60 min at room temperature.

For arginyltransferase (Ate1) kinetics measurements Aβ peptides were dissolved in 10% NH_4_OH in a concentration of 1 mM and incubated for 60 min at room temperature. The solution was frozen at -196°C and lyophilized to remove the solvent. Dried peptides were resuspended in reaction buffer. Only freshly prepared peptide solutions were used for all experiments.

### *In vitro* Transcription-Translation-Degradation assay

The TNT T7 Coupled Reticulocyte Lysate System (Promega) was used to carry out transcription-translation-degradation assays as described elsewhere [24]. Reaction samples were prepared according to the manufacturer’s instruction. Aβ peptides or equivalent amount of DMSO (control) were added to the samples. Nascent proteins in reticulocyte lysate were pulse-labeled with L-[^35^S]-methionine (10.2 mCi/ml, 1000 Ci/mmol, PerkinElmer) for 30 min at 30 °C, in the total volume of 17 μl. The labeling was quenched by the addition of 6.2 μl of chase medium (0.8 mg/ml cycloheximide, 8.3 mM unlabeled methionine, 4.2 mM ATP, 66.7 mM phosphocreatine, 0.3 mg/ml creatine kinase, 8.3 mM MgCl_2_, Aβ peptides or DMSO in required concentration). Samples were taken at indicated time points of a chase and the reactions were terminated by the addition of 80 μl of TSD buffer (1% SDS, 5 mM dithiothreitol (DTT), 50 mM Tris-HCl, pH 7.4) and snap-freezing in liquid nitrogen. Samples were then heated at 95°C for 10 min, diluted with 1 ml of TNN buffer (0.5% NP-40, 0.25 M NaCl, 5 mM EDTA, 50 mM Tris-HCl, pH 7.4), containing the “complete protease inhibitor mixture” (Roche), clarified by centrifugation at 15000 g for 5 min and immunoprecipitated using 5 μl of anti-flag M2 Magnetic Beads (Sigma). The samples were incubated with rocking at 4°C for 3 h, followed by 3 washes in TNN buffer, one wash in 10 mM Tris-HCl (pH 8.5), and eluted in 20 μl of SDS-sample buffer. Samples were then heated at 95°C for 10 min and fractionated by 10% SDS-PAGE, followed by autoradiography, using Typhoon FLA 9500 (GE Healthcare), and quantification, using ImageJ.

### Cell culture

Mouse neuroblastoma Neuro-2a cells were cultured at 37°C in 5% CO_2_ in DMEM medium supplemented with 10% fetal bovine serum (FBS, Invitrogen), 100 units/ml penicillin (Invitrogen), 0.1 mg/ml streptomycin (Invitrogen) and 2 mM glutamine (PanEko). For differentiation cells were grown in the medium containing 1% FBS for 2 days. The medium was replaced with a serum-free medium prior to adding amyloid peptides.

### Generation of Ate1 knockout Neuro-2a cells

Mouse neuroblastoma Ate1-lacking Neuro-2a cells were generated using the CRISPR/Cas9 system and were described previously [71].

### Flow cytometry

Flow cytometry analysis was performed to determine the percent of apoptotic cells and cells with damaged membrane by double staining with Pacific Blue conjugated Annexin-V (Molecular Probes) and propidium iodide (PI; Sigma). The cells were first washed with PBS at 4°C and resuspended in 0.1 ml (1×10^6^ cells/ml) of buffer-A (10 mM Hepes, 140 mM NaCl, 2.5 mM CaCl_2_, pH 7.4). Then, they were incubated with 5 μl of Pacific Blue-conjugated Annexin V (Ex/Em 410/455 nm) for 15 min at room temperature in darkness. 400 μl of buffer-A was added and cells were incubated with 10 μg/ml PI (Ex/Em 493/632 nm) for 1–2 min before analysis in a BD LSRFortessa flow cytometer (BD Biosciences). Data were analyzed using FlowJo software (Tree Star, Inc). The Annexin-V positive and PI negative cells were considered early apoptotic. Cells in subpopulations were expressed as a percentage of the total number of cells. The experiments were repeated thrice with triplicates and values were expressed as mean ± SD.

### Ate1 expression and purification

Mouse Ate1 isoforms (Ate1^1B7A^ and Ate1^1A7A^) were expressed and purified as described previously [72] with modifications. An overnight culture of transformed Rosetta^TM^(DE3)pLysS *E. coli* cells was split 1:100 into 400 ml of LB medium supplemented with standard concentrations of ampicillin and chloramphenicol, followed by growth at 37°C for 1-2 hours until A_600_ of ∼0.7. Expression was induced with 0.5 mM IPTG (isopropyl b-D-thiogalactoside), the culture was cold shocked on ice for 30 min, and then growth at 22°C for 15 h. For Ate1 purification, cells were collected by centrifuging at 4000 rpm for 20 min, resuspended in lysis buffer (10% glycerol, 0.05% Nonidet P-40, 500 mM NaCl, 20 mM imidazole, 5 mM β-mercaptoethanol, 50 mM Na_2_HPO_4_/NaH_2_PO_4_, pH 8.0) containing 1 mg/ml of lysozyme and “complete protease inhibitor mixture” (Roche), followed by brief sonication. The lysate was centrifuge at 12000 g for 20 min, 4°C and incubated for 1 h at 4°C with Ni-NTA agarose (GE Healthcare) equilibrated with the lysis buffer. The resin was washed three times with the lysis buffer containing 50 mM imidazole. Proteins were eluted with lysis buffer containing 250 mM imidazole, followed by overnight dialysis at 4°C against 50 mM Na_2_HPO_4_/NaH_2_PO_4_, pH 7.5 containing 10% glycerol, 0.05% Nonidet P-40, 300 mM NaCl, 5 mM β-mercaptoethanol. The tagged Ub moiety was cleaved off during dialysis by incubation with Usp2-cc (1:100), a deubiquitylating enzyme that has been expressed and purified as previously described [73]. To eliminate His-tagged Ub and Usp2-cc proteins were incubated with Ni-NTA agarose equilibrated with the dialysis buffer, followed by washing in dialysis buffer containing 500 mM NaCl. Untagged Ate1 isoforms were eluted with dialysis buffer containing 500 mM NaCl, 50 mM imidazole and then dialyzed against 50 mM Na_2_HPO_4_/NaH_2_PO_4_, pH 7.5 containing 150 mM NaCl, 5 mM MgCl_2_, 2 mM DTT, 20% glycerol for 2 h at 4°C. Glycerol was added to the dialyzed samples to a final concentration of 50%. Purified Ate1 isoforms were kept at -80°C. The estimated purity of each protein was >90%.

### Enzyme kinetic activity

The enzymatic kinetics was measured using a MicroCal PEAQ-ITC (Malvern). Experiments were carry out at 37°C in 50 mM Hepes, pH 7.5, 30 mM KCl, 5 mM MgCl_2_, 1 mM DTT and 200 μM L-arginine. Single aliquot (8 μl, 16s) of the 5 mM BSA or 1.25 mM Aβ peptides was injected from the syringe into the 0.2 ml cell containing 1 μM of Ate1, 50 μM ATP, 100 μM total *E. coli* tRNA (Sigma), 1 μM total *E. coli* aminoacyl-tRNA synthetases (RS). To evaluate inhibition effect of Aβ peptides on the RS enzymatic activity 8 μl of 10 mM ATP was injected into mixture of 1 μM RS and 1 mM tRNA in the presence and absence of inhibitor (100 μM Aβ). The resulting kinetic curves were fitted using MicroCal PEAQ-ITC Analysis Software (Malvern). Michaelis constants (K_M_), catalytic rate constants for substrate conversion (k_cat_) and inhibition constants (K_I_) were determined by non-linear least squares. To calculate the K_I_ values, the kinetic parameters for RS obtained in the absence of amyloid peptides were used.

### ELISA

All antibodies used in ELISA assays are listed in Table 3. 96-well plate (Thermo Scientific Nunc MaxiSorp Surface) was coated overnight with 50 μl of capture antibody (2 μg/ml) followed by washing with phosphate-buffered saline containing 0.05% Tween (PBST) and incubation with 100 μl of blocking buffer for 2 h. One hundred microliters of 1 μM Aβ was added to the wells for 90 min at 37°C. After washing, the wells were additionally incubated with buffer (30 mM KCl, 5 mM MgCl_2_, 15 μM Arg, 1mM ATP, 50 mM Hepes, pH 7.5), Arg-tRNA or Ate1 for 90 min at 37°C. To synthetize Arg-tRNA total tRNA was first incubated with RS (1000 u/ml) and 50 mM Hepes, pH 7.5 containing 50 μM Arg, 2.5 mM ATP, 2 mM DTT, 5mM MgCl_2_ and 30mM KCl at 37°C for 60 min, then purified away from the proteins using phenol/chlorophorm extraction. All wells were washed three times with 200 μl of PBST. 100 μl of detection antibody (0.7 μg/ml) was added to each well and incubated for 2 h. After washing, 100 μl of horseradish peroxidase (HRP)-conjugated antibody diluted at the optimal concentration in blocking buffer was incubated with samples for 1 h. For detection OPD (o-phenylenediamine dihydrochloride, Thermo Scientific) was used according to manufacturer’s instruction. Absorbance values were measured at 450 nm by a Multiskan™ GO Microplate Spectrophotometer (Thermo Scientific).

**Table 3:** Antibodies used in ELISA assays.

**Antibody**	**Host**	**Manufacturer**	**Dilution**
**Capture Antibody**			
**anti-Aβ_1-17_ (DE2B2)**	Mouse monoclonal	Thermo Fisher Scientific, USA cat. #MA1-24966	1:500
**anti-Aβ_36-42_**	Rabbit polyclonal	Thermo Fisher Scientific, USA cat. #44-344	1:500
**Detection Antibody**			
**anti-Aβ_36-42_**	Rabbit polyclonal	Thermo Fisher Scientific, USA cat. #44-344	1:1500
**anti-Aβ_1-17_ (DE2B2)**	Mouse monoclonal	Thermo Fisher Scientific, USA cat. #MA1-24966	1:1500
**Secondary HRP-conjugated Antibody**			
**anti-rabbit**	Donkey polyclonal	Novex, USA cat. #A16035	1:10000
**anti-mouse**	Rabbit polyclonal	Imtek, RF cat. #RAM Iss	1:10000

### Statistical analysis

The data are shown as the mean ± standard deviation at least of three independent experiments. The differences among the groups were analyzed using One Way ANOVA with post-hoc Tukey HSD (Honestly Significant Difference) test.

## Supplementary Material

Supplementary Figures

## References

[1] Walsh DM, Selkoe DJ. A critical appraisal of the pathogenic protein spread hypothesis of neurodegeneration. Nat Rev Neurosci. 2016; 17:251–60. 10.1038/nrn.2016.13 26988744 PMC6701169

[2] Khachaturian ZS. Diagnosis of Alzheimer’s disease. Arch Neurol. 1985; 42:1097–105. 10.1001/archneur.1985.04060100083029 2864910

[3] Mirra SS, Hart MN, Terry RD. Making the diagnosis of Alzheimer’s disease. A primer for practicing pathologists. Arch Pathol Lab Med. 1993; 117:132–44. 8427562

[4] Ji XR, Cheng KC, Chen YR, Lin TY, Cheung CH, Wu CL, Chiang HC. Dysfunction of different cellular degradation pathways contributes to specific β-amyloid42-induced pathologies. FASEB J. 2018; 32:1375–87. 10.1096/fj.201700199RR 29127191

[5] Ntsapi C, Lumkwana D, Swart C, du Toit A, Loos B. New Insights Into Autophagy Dysfunction Related to Amyloid Beta Toxicity and Neuropathology in Alzheimer’s Disease. 1st ed. International Review of Cell and Molecular Biology. Elsevier Inc.; 2017. 321–361 p. 10.1016/bs.ircmb.2017.07.002 29413893

[6] Penke B, Bogár F, Fülöp L. β-Amyloid and the Pathomechanisms of Alzheimer’s Disease: A Comprehensive View. Molecules. 2017; 22:1692. 10.3390/molecules22101692 28994715 PMC6151811

[7] Keller JN, Hanni KB, Markesbery WR. Impaired proteasome function in Alzheimer’s disease. J Neurochem. 2000; 75:436–39. 10.1046/j.1471-4159.2000.0750436.x 10854289

[8] López Salon M, Morelli L, Castaño EM, Soto EF, Pasquini JM. Defective ubiquitination of cerebral proteins in Alzheimer’s disease. J Neurosci Res. 2000; 62:302–10. 10.1002/1097-4547(20001015)62:2<302::AID-JNR15>3.0.CO;2-L 11020223

[9] Reiss AB, Arain HA, Stecker MM, Siegart NM, Kasselman LJ. Amyloid toxicity in Alzheimer’s disease. Rev Neurosci. 2018; 29:613–27. 10.1515/revneuro-2017-0063 29447116

[10] Mitkevich VA, Petrushanko IY, Yegorov YE, Simonenko OV, Vishnyakova KS, Kulikova AA, Tsvetkov PO, Makarov AA, Kozin SA. Isomerization of Asp7 leads to increased toxic effect of amyloid-β42 on human neuronal cells. Cell Death Dis. 2013; 4:e939. 10.1038/cddis.2013.492 24287700 PMC3847340

[11] Farizatto KL, Ikonne US, Almeida MF, Ferrari MF, Bahr BA. Aβ42-mediated proteasome inhibition and associated tau pathology in hippocampus are governed by a lysosomal response involving cathepsin B: evidence for protective crosstalk between protein clearance pathways. PLoS One. 2017; 12:e0182895. 10.1371/journal.pone.0182895 28797057 PMC5552263

[12] Cecarini V, Bonfili L, Cuccioloni M, Mozzicafreddo M, Rossi G, Buizza L, Uberti D, Angeletti M, Eleuteri AM. Crosstalk between the ubiquitin-proteasome system and autophagy in a human cellular model of Alzheimer’s disease. Biochim Biophys Acta. 2012; 1822:1741–51. 10.1016/j.bbadis.2012.07.015 22867901

[13] Morozov AV, Kulikova AA, Astakhova TM, Mitkevich VA, Burnysheva KM, Adzhubei AA, Erokhov PA, Evgen’ev MB, Sharova NP, Karpov VL, Makarov AA. Amyloid-β Increases Activity of Proteasomes Capped with 19S and 11S Regulators. J Alzheimers Dis. 2016; 54:763–76. 27567864 10.3233/JAD-160491

[14] Orre M, Kamphuis W, Dooves S, Kooijman L, Chan ET, Kirk CJ, Dimayuga Smith V, Koot S, Mamber C, Jansen AH, Ovaa H, Hol EM. Reactive glia show increased immunoproteasome activity in Alzheimer’s disease. Brain. 2013; 136:1415–31. 10.1093/brain/awt083 23604491

[15] Eisenach PA, Schikora F, Posern G. Inhibition of arginyltransferase 1 induces transcriptional activity of myocardin-related transcription factor A (MRTF-A) and promotes directional migration. J Biol Chem. 2014; 289:35376–87. 10.1074/jbc.M114.578674 25381249 PMC4271223

[16] Chen SJ, Wu X, Wadas B, Oh JH, Varshavsky A. An N-end rule pathway that recognizes proline and destroys gluconeogenic enzymes. Science. 2017; 355:eaal3655. 10.1126/science.aal3655 28126757 PMC5457285

[17] Varshavsky A. The N-end rule pathway and regulation by proteolysis. Protein Sci. 2011; 20:1298–345. 10.1002/pro.666 21633985 PMC3189519

[18] Kim JM, Hwang CS. Crosstalk between the Arg/N-end and Ac/N-end rule. Cell Cycle. 2014; 13:1366–67. 10.4161/cc.28751 24698805 PMC4050130

[19] Lee KE, Heo JE, Kim JM, Hwang CS. N-Terminal Acetylation-Targeted N-End Rule Proteolytic System: The Ac/N-End Rule Pathway. Mol Cells. 2016; 39:169–78. 10.14348/molcells.2016.2329 26883906 PMC4794598

[20] Park SE, Kim JM, Seok OH, Cho H, Wadas B, Kim SY, Varshavsky A, Hwang CS. Control of mammalian G protein signaling by N-terminal acetylation and the N-end rule pathway. Science. 2015; 347:1249–52. 10.1126/science.aaa3844 25766235 PMC4748709

[21] Hwang CS, Shemorry A, Varshavsky A. N-terminal acetylation of cellular proteins creates specific degradation signals. Science. 2010; 327:973–77. 10.1126/science.1183147 20110468 PMC4259118

[22] Kim HK, Kim RR, Oh JH, Cho H, Varshavsky A, Hwang CS. The N-terminal methionine of cellular proteins as a degradation signal. Cell. 2014; 156:158–69. 10.1016/j.cell.2013.11.031 24361105 PMC3988316

[23] Piatkov KI, Oh JH, Liu Y, Varshavsky A. Calpain-generated natural protein fragments as short-lived substrates of the N-end rule pathway. Proc Natl Acad Sci USA. 2014; 111:E817–26. 10.1073/pnas.1401639111 24550490 PMC3948289

[24] Brower CS, Piatkov KI, Varshavsky A. Neurodegeneration-associated protein fragments as short-lived substrates of the N-end rule pathway. Mol Cell. 2013; 50:161–71. 10.1016/j.molcel.2013.02.009 23499006 PMC3640747

[25] Kim MK, Oh SJ, Lee BG, Song HK. Structural basis for dual specificity of yeast N-terminal amidase in the N-end rule pathway. Proc Natl Acad Sci USA. 2016; 113:12438–43. 10.1073/pnas.1612620113 27791147 PMC5098633

[26] Wang H, Piatkov KI, Brower CS, Varshavsky A. Glutamine-specific N-terminal amidase, a component of the N-end rule pathway. Mol Cell. 2009; 34:686–95. 10.1016/j.molcel.2009.04.032 19560421 PMC2749074

[27] Grigoryev S, Stewart AE, Kwon YT, Arfin SM, Bradshaw RA, Jenkins NA, Copeland NG, Varshavsky A. A mouse amidase specific for N-terminal asparagine. The gene, the enzyme, and their function in the N-end rule pathway. J Biol Chem. 1996; 271:28521–32. 10.1074/jbc.271.45.28521 8910481

[28] Liu YJ, Liu C, Chang Z, Wadas B, Brower CS, Song ZH, Xu ZL, Shang YL, Liu WX, Wang LN, Dong W, Varshavsky A, Hu RG, Li W. Degradation of the Separase-cleaved Rec8, a Meiotic Cohesin Subunit, by the N-end Rule Pathway. J Biol Chem. 2016; 291:7426–38. 10.1074/jbc.M116.714964 26858254 PMC4817174

[29] Wadas B, Piatkov KI, Brower CS, Varshavsky A. Analyzing N-terminal Arginylation through the Use of Peptide Arrays and Degradation Assays. J Biol Chem. 2016; 291:20976–92. 10.1074/jbc.M116.747956 27510035 PMC5076509

[30] Tasaki T, Sriram SM, Park KS, Kwon YT. The N-end rule pathway. Annu Rev Biochem. 2012; 81:261–89. 10.1146/annurev-biochem-051710-093308 22524314 PMC3610525

[31] Wang J, Han X, Saha S, Xu T, Rai R, Zhang F, Wolf YI, Wolfson A, Yates JR 3rd, Kashina A. Arginyltransferase is an ATP-independent self-regulating enzyme that forms distinct functional complexes in vivo. Chem Biol. 2011; 18:121–30. 10.1016/j.chembiol.2010.10.016 21276945 PMC3031169

[32] Soffer RL. Enzymatic modification of proteins. II. Purification and properties of the arginyl transfer ribonucleic acid-protein transferase from rabbit liver cytoplasm. J Biol Chem. 1970; 245:731–37. 5416661

[33] Janssen JC, Beck JA, Campbell TA, Dickinson A, Fox NC, Harvey RJ, Houlden H, Rossor MN, Collinge J. Early onset familial Alzheimer’s disease: mutation frequency in 31 families. Neurology. 2003; 60:235–39. 10.1212/01.WNL.0000042088.22694.E3 12552037

[34] Istrate AN, Kozin SA, Zhokhov SS, Mantsyzov AB, Kechko OI, Pastore A, Makarov AA, Polshakov VI. Interplay of histidine residues of the Alzheimer’s disease Aβ peptide governs its Zn-induced oligomerization. Sci Rep. 2016; 6:21734. 10.1038/srep21734 26898943 PMC4761979

[35] Ono K, Condron MM, Teplow DB. Effects of the English (H6R) and Tottori (D7N) familial Alzheimer disease mutations on amyloid beta-protein assembly and toxicity. J Biol Chem. 2010; 285:23186–97. 10.1074/jbc.M109.086496 20452980 PMC2906312

[36] Piatkov KI, Brower CS, Varshavsky A. The N-end rule pathway counteracts cell death by destroying proapoptotic protein fragments. Proc Natl Acad Sci USA. 2012; 109:E1839–47. 10.1073/pnas.1207786109 22670058 PMC3390858

[37] Wang J, Han X, Wong CC, Cheng H, Aslanian A, Xu T, Leavis P, Roder H, Hedstrom L, Yates JR 3rd, Kashina A. Arginyltransferase ATE1 catalyzes midchain arginylation of proteins at side chain carboxylates in vivo. Chem Biol. 2014; 21:331–37. 10.1016/j.chembiol.2013.12.017 24529990 PMC4010198

[38] Chen WT, Hong CJ, Lin YT, Chang WH, Huang HT, Liao JY, Chang YJ, Hsieh YF, Cheng CY, Liu HC, Chen YR, Cheng IH. Amyloid-beta (Aβ) D7H mutation increases oligomeric Aβ42 and alters properties of Aβ-zinc/copper assemblies. PLoS One. 2012; 7:e35807. 10.1371/journal.pone.0035807 22558227 PMC3340413

[39] Kumar-Singh S, Julliams A, Nuydens R, Ceuterick C, Labeur C, Serneels S, Vennekens K, Van Osta P, Geerts H, De Strooper B, Van Broeckhoven C. In vitro studies of Flemish, Dutch, and wild-type β-amyloid provide evidence for two-staged neurotoxicity. Neurobiol Dis. 2002; 11:330–40. 10.1006/nbdi.2002.0529 12505425

[40] Grabowski TJ, Cho HS, Vonsattel JP, Rebeck GW, Greenberg SM. Novel amyloid precursor protein mutation in an Iowa family with dementia and severe cerebral amyloid angiopathy. Ann Neurol. 2001; 49:697–705. 10.1002/ana.1009 11409420

[41] Nilsberth C, Westlind-Danielsson A, Eckman CB, Condron MM, Axelman K, Forsell C, Stenh C, Luthman J, Teplow DB, Younkin SG, Näslund J, Lannfelt L. The ‘Arctic’ APP mutation (E693G) causes Alzheimer’s disease by enhanced Abeta protofibril formation. Nat Neurosci. 2001; 4:887–93. 10.1038/nn0901-887 11528419

[42] Zhang L, Trushin S, Christensen TA, Tripathi U, Hong C, Geroux RE, Howell KG, Poduslo JF, Trushina E. Differential effect of amyloid beta peptides on mitochondrial axonal trafficking depends on their state of aggregation and binding to the plasma membrane. Neurobiol Dis. 2018; 114:1–16. 10.1016/j.nbd.2018.02.003 29477640 PMC5926207

[43] Rajasekhar K, Chakrabarti M, Govindaraju T. Function and toxicity of amyloid beta and recent therapeutic interventions targeting amyloid beta in Alzheimer’s disease. Chem Commun (Camb). 2015; 51:13434–50. 10.1039/C5CC05264E 26247608

[44] Kayed R, Lasagna-Reeves CA. Molecular mechanisms of amyloid oligomers toxicity. J Alzheimers Dis. 2013 (Suppl 1); 33:S67–78. 22531422 10.3233/JAD-2012-129001

[45] Hu RG, Brower CS, Wang H, Davydov IV, Sheng J, Zhou J, Kwon YT, Varshavsky A. Arginyltransferase, its specificity, putative substrates, bidirectional promoter, and splicing-derived isoforms. J Biol Chem. 2006; 281:32559–73. 10.1074/jbc.M604355200 16943202

[46] Rai R, Kashina A. Identification of mammalian arginyltransferases that modify a specific subset of protein substrates. Proc Natl Acad Sci USA. 2005; 102:10123–28. 10.1073/pnas.0504500102 16002466 PMC1173364

[47] Kwon YT, Kashina AS, Varshavsky A. Alternative splicing results in differential expression, activity, and localization of the two forms of arginyl-tRNA-protein transferase, a component of the N-end rule pathway. Mol Cell Biol. 1999; 19:182–93. 10.1128/MCB.19.1.182 9858543 PMC83877

[48] Lian L, Suzuki A, Hayes V, Saha S, Han X, Xu T, Yates JR 3rd, Poncz M, Kashina A, Abrams CS. Loss of ATE1-mediated arginylation leads to impaired platelet myosin phosphorylation, clot retraction, and in vivo thrombosis formation. Haematologica. 2014; 99:554–60. 10.3324/haematol.2013.093047 24293517 PMC3943321

[49] Saha S, Mundia MM, Zhang F, Demers RW, Korobova F, Svitkina T, Perieteanu AA, Dawson JF, Kashina A. Arginylation regulates intracellular actin polymer level by modulating actin properties and binding of capping and severing proteins. Mol Biol Cell. 2010; 21:1350–61. 10.1091/mbc.e09-09-0829 20181827 PMC2854093

[50] Wang YM, Ingoglia NA. N-terminal arginylation of sciatic nerve and brain proteins following injury. Neurochem Res. 1997; 22:1453–59. 10.1023/A:1021998227237 9357010

[51] Chakraborty G, Ingoglia NA. N-terminal arginylation and ubiquitin-mediated proteolysis in nerve regeneration. Brain Res Bull. 1993; 30:439–45. 10.1016/0361-9230(93)90276-H 8384516

[52] Xu NS, Chakraborty G, Hassankhani A, Ingoglia NA. N-terminal arginylation of proteins in explants of injured sciatic nerves and embryonic brains of rats. Neurochem Res. 1993; 18:1117–23. 10.1007/BF00978361 8255362

[53] Lee MJ, Kim DE, Zakrzewska A, Yoo YD, Kim SH, Kim ST, Seo JW, Lee YS, Dorn GW 2nd, Oh U, Kim BY, Kwon YT. Characterization of arginylation branch of N-end rule pathway in G-protein-mediated proliferation and signaling of cardiomyocytes. J Biol Chem. 2012; 287:24043–52. 10.1074/jbc.M112.364117 22577142 PMC3390678

[54] Lee MJ, Tasaki T, Moroi K, An JY, Kimura S, Davydov IV, Kwon YT. RGS4 and RGS5 are in vivo substrates of the N-end rule pathway. Proc Natl Acad Sci USA. 2005; 102:15030–35. 10.1073/pnas.0507533102 16217033 PMC1257735

[55] Saha S, Wang J, Buckley B, Wang Q, Lilly B, Chernov M, Kashina A. Small molecule inhibitors of arginyltransferase regulate arginylation-dependent protein degradation, cell motility, and angiogenesis. Biochem Pharmacol. 2012; 83:866–73. 10.1016/j.bcp.2012.01.012 22280815 PMC3288401

[56] Kwon YT, Kashina AS, Davydov IV, Hu RG, An JY, Seo JW, Du F, Varshavsky A. An essential role of N-terminal arginylation in cardiovascular development. Science. 2002; 297:96–99. 10.1126/science.1069531 12098698

[57] Kaji H, Hara H, Lamon KD. Fixation of cellular aging processes by SV40 virus transformation. Mech Ageing Dev. 1980; 12:197–209. 10.1016/0047-6374(80)90095-0 6245312

[58] Kumar A, Birnbaum MD, Patel DM, Morgan WM, Singh J, Barrientos A, Zhang F. Posttranslational arginylation enzyme Ate1 affects DNA mutagenesis by regulating stress response. Cell Death Dis. 2016; 7:e2378–2378. 10.1038/cddis.2016.284 27685622 PMC5059882

[59] Wang J, Han X, Leu NA, Sterling S, Kurosaka S, Fina M, Lee VM, Dong DW, Yates JR 3rd, Kashina A. Protein arginylation targets alpha synuclein, facilitates normal brain health, and prevents neurodegeneration. Sci Rep. 2017; 7:11323. 10.1038/s41598-017-11713-z 28900170 PMC5595787

[60] Yoo YD, Mun SR, Ji CH, Sung KW, Kang KY, Heo AJ, Lee SH, An JY, Hwang J, Xie XQ, Ciechanover A, Kim BY, Kwon YT. N-terminal arginylation generates a bimodal degron that modulates autophagic proteolysis. Proc Natl Acad Sci USA. 2018; 115:E2716–24. 10.1073/pnas.1719110115 29507222 PMC5866579

[61] Ji CH, Kwon YT. Crosstalk and Interplay between the Ubiquitin-Proteasome System and Autophagy. Mol Cells. 2017; 40:441–49. 10.14348/molcells.2017.0115 28743182 PMC5547213

[62] Cha-Molstad H, Kwon YT, Kim BY. Amino-terminal arginylation as a degradation signal for selective autophagy. BMB Rep. 2015; 48:487–88. 10.5483/BMBRep.2015.48.9.176 26303972 PMC4641230

[63] Jiang Y, Lee J, Lee JH, Lee JW, Kim JH, Choi WH, Yoo YD, Cha-Molstad H, Kim BY, Kwon YT, Noh SA, Kim KP, Lee MJ. The arginylation branch of the N-end rule pathway positively regulates cellular autophagic flux and clearance of proteotoxic proteins. Autophagy. 2016; 12:2197–212. 10.1080/15548627.2016.1222991 27560450 PMC5103369

[64] Baldassarro VA, Marchesini A, Giardino L, Calzà L. Vulnerability of primary neurons derived from Tg2576 Alzheimer mice to oxygen and glucose deprivation: role of intraneuronal amyloid-β accumulation and astrocytes. Dis Model Mech. 2017; 10:671–78. 10.1242/dmm.028001 28237964 PMC5451168

[65] Harwell CS, Coleman MP. Synaptophysin depletion and intraneuronal Aβ in organotypic hippocampal slice cultures from huAPP transgenic mice. Mol Neurodegener. 2016; 11:44. 10.1186/s13024-016-0110-7 27287430 PMC4903008

[66] Billings LM, Oddo S, Green KN, McGaugh JL, LaFerla FM. Intraneuronal Abeta causes the onset of early Alzheimer’s disease-related cognitive deficits in transgenic mice. Neuron. 2005; 45:675–88. 10.1016/j.neuron.2005.01.040 15748844

[67] Lopez EM, Bell KF, Ribeiro-da-Silva A, Cuello AC. Early changes in neurons of the hippocampus and neocortex in transgenic rats expressing intracellular human a-beta. J Alzheimers Dis. 2004; 6:421–31. 15345813 10.3233/jad-2004-6410

[68] Knauer MF, Soreghan B, Burdick D, Kosmoski J, Glabe CG, Lee JM. Intracellular accumulation and resistance to degradation of the Alzheimer amyloid A4/beta protein. Proc Natl Acad Sci USA. 1992; 89:7437–41. 10.1073/pnas.89.16.7437 1502155 PMC49725

[69] Brower CS, Rosen CE, Jones RH, Wadas BC, Piatkov KI, Varshavsky A. Liat1, an arginyltransferase-binding protein whose evolution among primates involved changes in the numbers of its 10-residue repeats. Proc Natl Acad Sci USA. 2014; 111:E4936–45. 10.1073/pnas.1419587111 25369936 PMC4246273

[70] Klein WL. Abeta toxicity in Alzheimer’s disease: globular oligomers (ADDLs) as new vaccine and drug targets. Neurochem Int. 2002; 41:345–52. 10.1016/S0197-0186(02)00050-5 12176077

[71] Kasu YA, Alemu S, Lamari A, Loew N, Brower CS. The N-termini of TAR DNA-binding protein-43 (TDP43) C-terminal fragments influence degradation, aggregation propensity and morphology. Mol Cell Biol. 2018; 38:e00243–18. 10.1128/MCB.00243-18 29987190 PMC6146831

[72] Hu RG, Wang H, Xia Z, Varshavsky A. The N-end rule pathway is a sensor of heme. Proc Natl Acad Sci USA. 2008; 105:76–81. 10.1073/pnas.0710568105 18162538 PMC2224235

[73] Catanzariti AM, Soboleva TA, Jans DA, Board PG, Baker RT. An efficient system for high-level expression and easy purification of authentic recombinant proteins. Protein Sci. 2004; 13:1331–39. 10.1110/ps.04618904 15096636 PMC2286746

