# Supplementary material for: Beta-amyloid induces apoptosis of neuronal cells by inhibition of the Arg/N-end rule pathway proteolytic activity
Source: Aging (Albany NY). 2019 Aug 24;11(16):6134–52. doi: 10.18632/aging.102177 (PMC6738421; doi:10.18632/aging.102177)
Supplement: Supplementary Figures [file aging-11-102177-s001.pdf]

SUPPLEMENTARY FIGURES

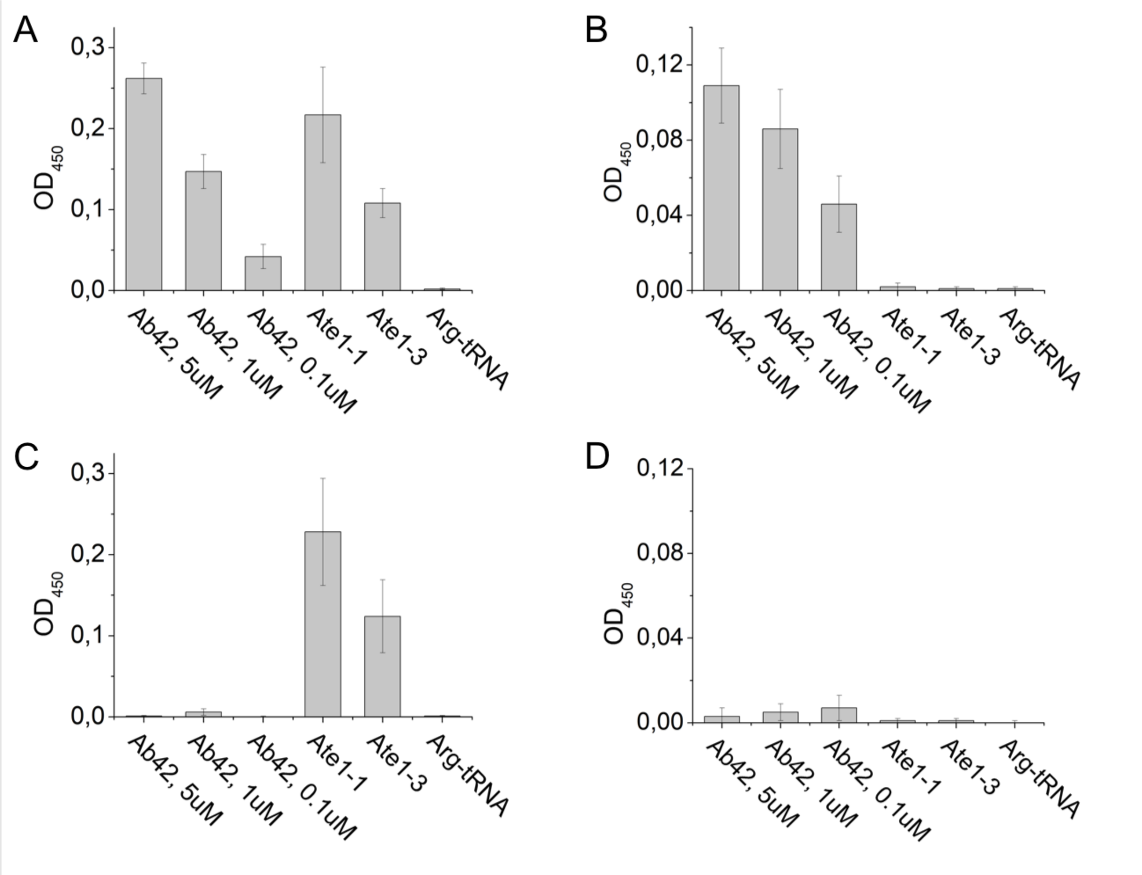

Supplementary Figure 1. Specificity of the anti-Aβ_{1-17} (**A**), anti-Aβ_{36-42} (**B**), anti-mouse (**C**) and anti-rabbit (**D**) antibodies to Aβ_{42}, Ate1 isoforms and tRNA. Specificity of antibodies was probed using the indirect ELISA method. 96-well plate (Thermo Scientific Nunc MaxiSorp Surface) was coated overnight with 50 µl of Aβ_{42}, 3 µM Ate1 or 15 µM tRNA followed by washing with phosphate-buffered saline containing 0.05% Tween (PBST) and incubation with 200 µl of blocking buffer for 2 h. 100 µl of antibodies diluted at the optimal concentration (Table 3) was added to each well and incubated for 2 h. For detection OPD (o-phenylenediamine dihydrochloride, Thermo Scientific) was used according to manufacturer's instruction. OD_{450} – optical density measured at 450 nm. Each value is the mean ± SD of at least three independent experiments.

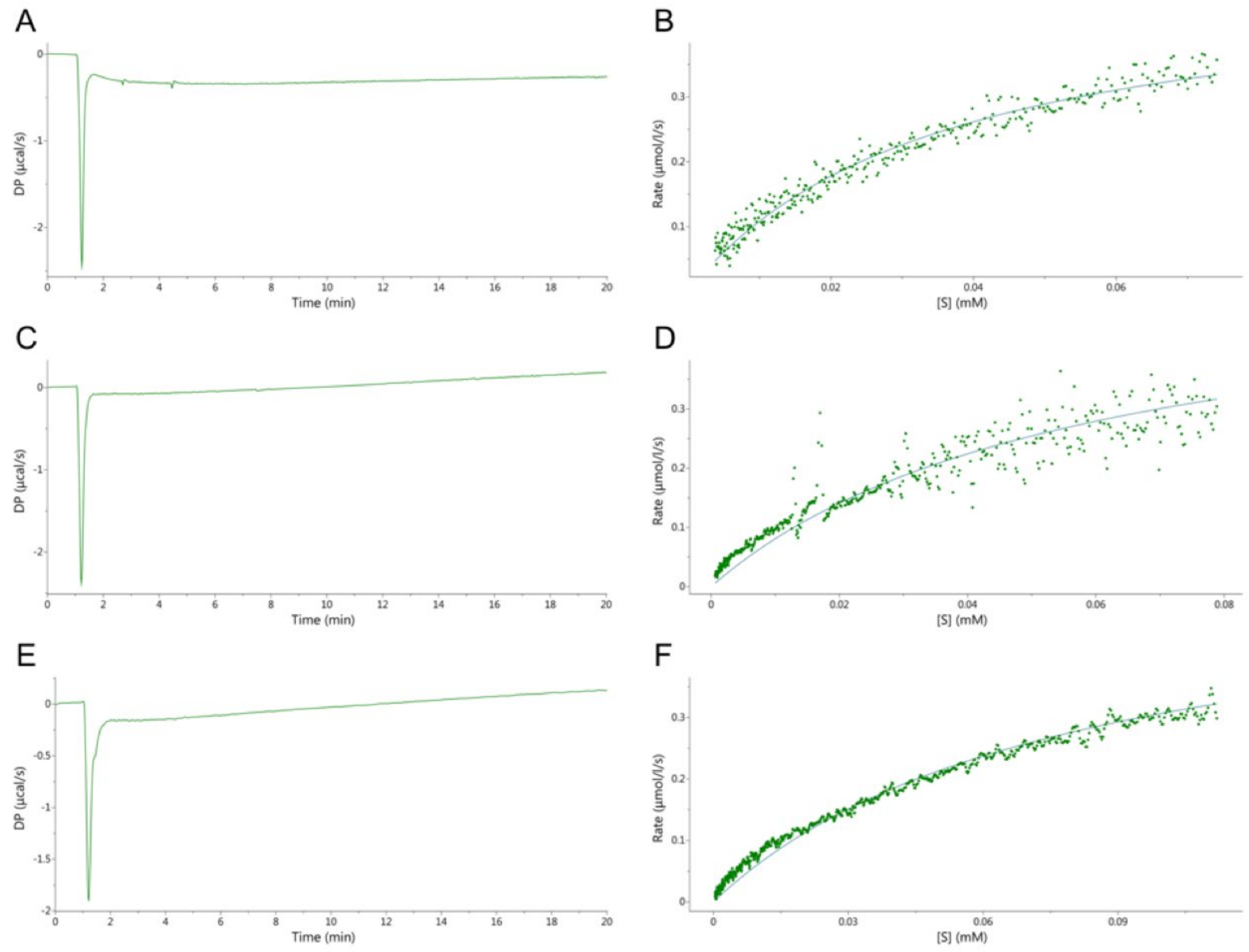

Supplementary Figure 2. Effect of Aβ on the ATP hydrolysis mediated by RS. (**A**) Raw thermal power obtained in the single injection ITC assay by injecting ATP (to 200 μM) into 1 μM RS and 1 mM tRNA at 37°C. (**B**) To determine RS kinetic parameters thermal power was converted to enzyme turnover and fitted by non-linear least squares. (**C**) and (**E**) same as (A) but in the presence of $A\beta_{42}$ and H6R-$A\beta_{42}$ in the calorimetric cell, respectively. Inhibition effect of $A\beta_{42}$ (**D**) and H6R-$A\beta_{42}$ (**F**) on ATP hydrolysis was evaluated using parameters of RS kinetic from (B) and non-linear least squares fitting.

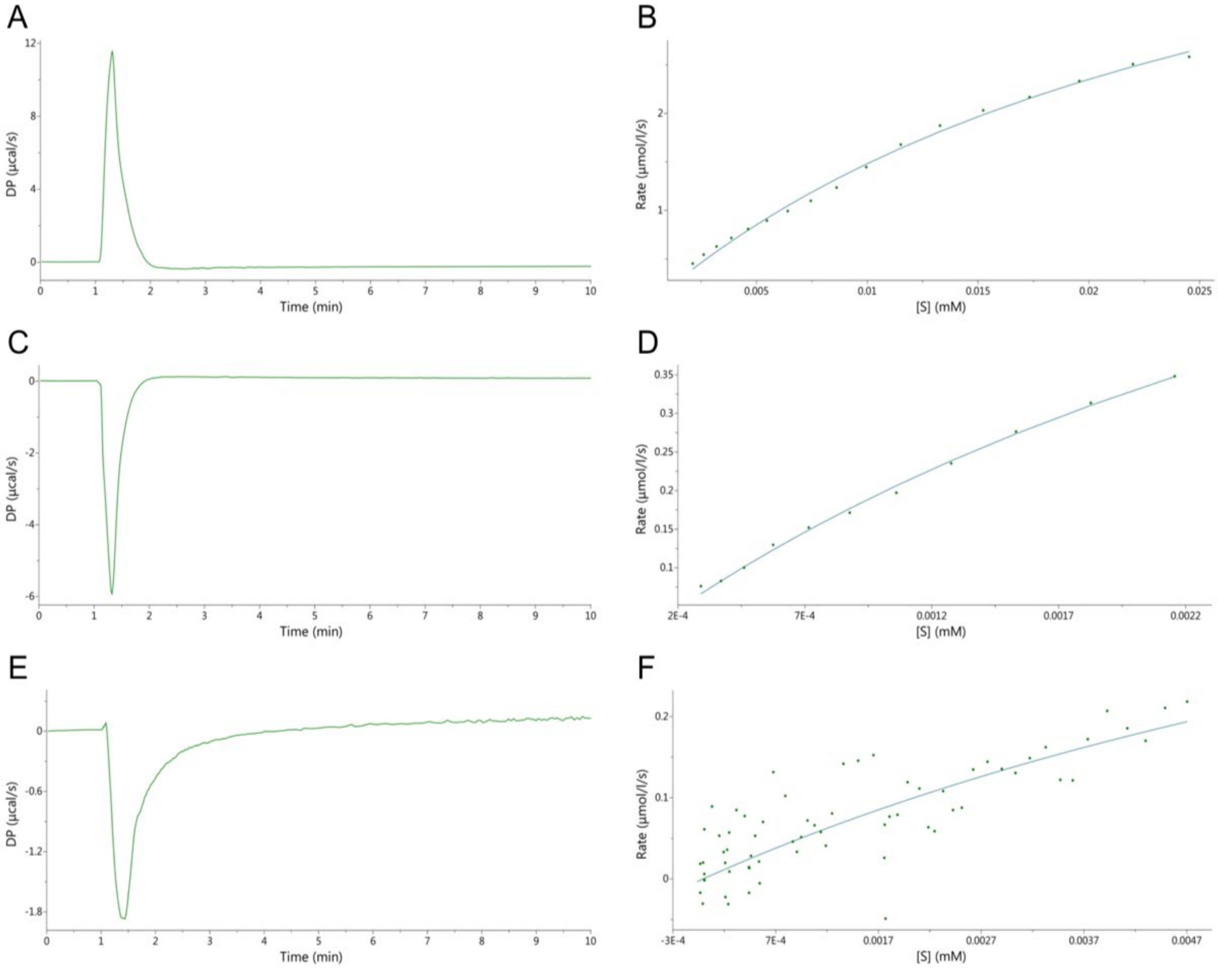

Supplementary Figure 3. Enzymatic kinetics of Ate1-1 with different substrates. Raw thermal power was obtained in the single injection ITC assay, performed at 37°C with injections of BSA (**A**), Aβ_{42} (**C**) or H6R-Aβ_{42} (**E**) into a mixture of Ate1-1, RS, and tRNA in the calorimetric cell. Kinetic curves for BSA (**B**), Aβ_{42} (**D**), and H6R-Aβ_{42} (**F**) were fitted to the Michaelis–Menten equation to determine the kinetic parameters of Ate1-1.

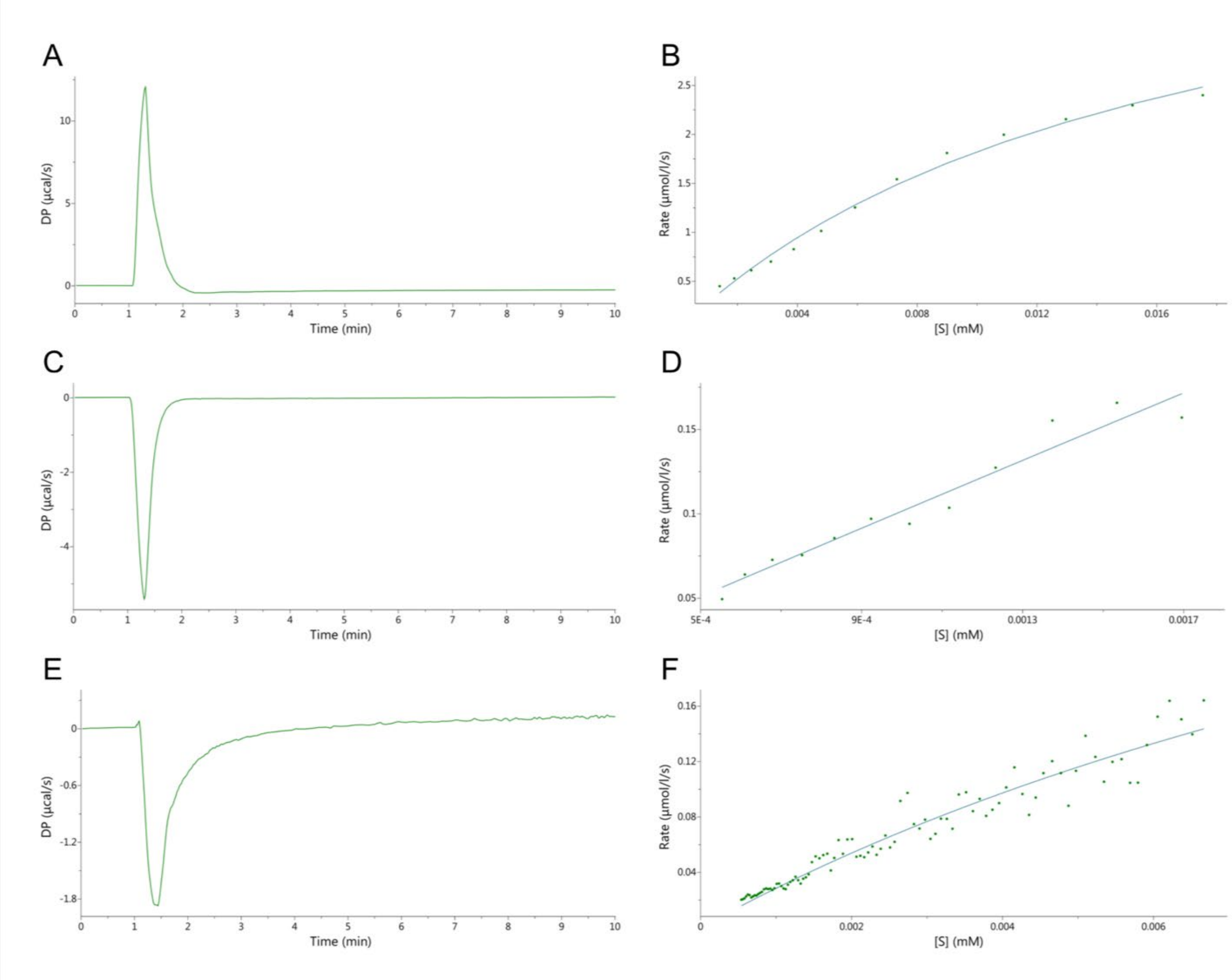

Supplementary Figure 4. Enzymatic kinetics of Ate1-3 with different substrates. Raw thermal power was obtained in the single injection ITC assay, performed at 37°C with injections of BSA (**A**), Aβ_{42} (**C**) or H6R-Aβ_{42} (**E**) into a mixture of Ate1-3, RS, and tRNA in the calorimetric cell. Kinetic curves for BSA (**B**), Aβ_{42} (**D**), and H6R-Aβ_{42} (**F**) were fitted to the Michaelis–Menten equation to determine the kinetic parameters of Ate1-3.

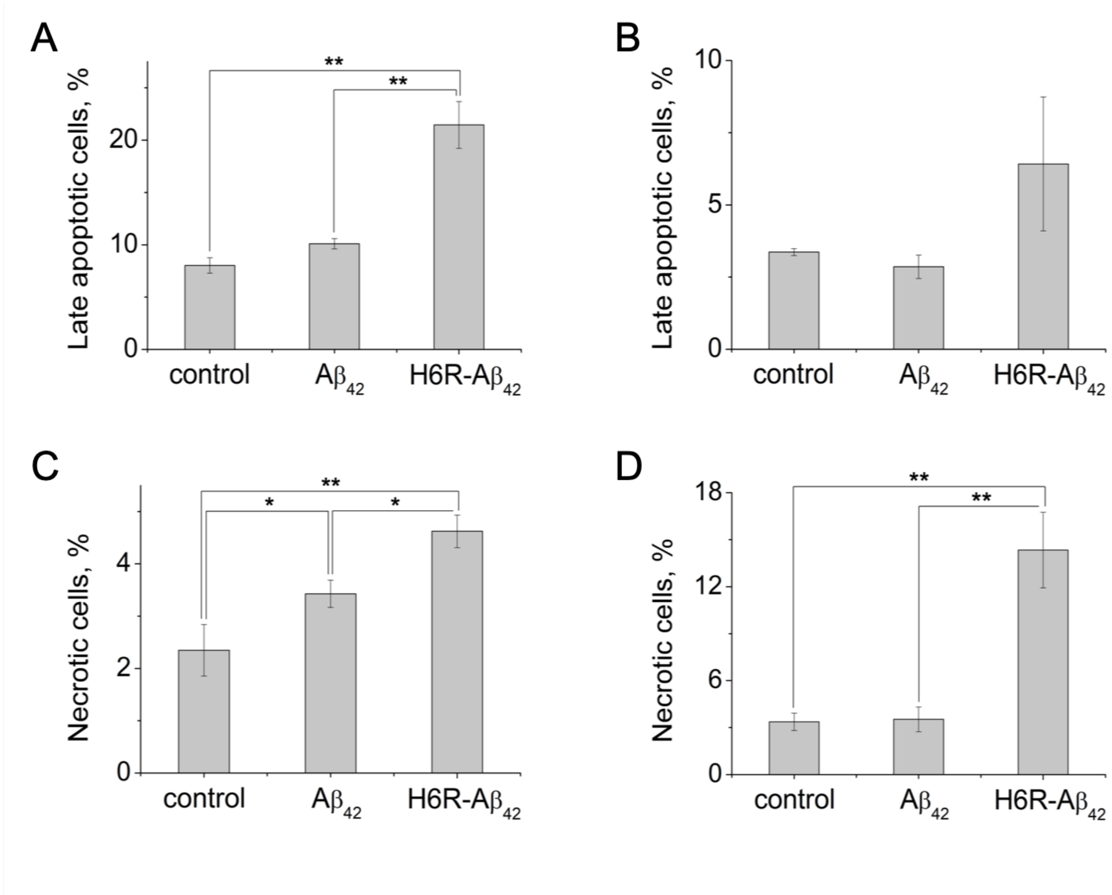

Supplementary Figure 5. Toxic effects of Aβ peptides (10 μM, 20 h) on differentiated Neuro-2a (**A, C**) cells and Ate1 knockout Neuro-2a cells (**B, D**). Cells stained by Annexin-V and propidium iodide (PI) are late apoptotic. The Annexin-V negative and PI positive cells were considered necrotic. Each value is expressed as a percentage of the total number of cells ± SD. The experiments were performed three times in triplicates; $*p < 0.05$, $**p < 0.01$.
